# Vactosertib enhances radiotherapy efficacy by modulating radiation-induced tumor microenvironment remodeling in breast cancer

**DOI:** 10.2478/raon-2026-0034

**Published:** 2026-06-26

**Authors:** Jiyoung Park, Jiwon Choi, Yhun Yhong Sheen

**Affiliations:** 1 College of Pharmacy, Ewha Womans University, Seodaemun-gu, Seoul, Republic of Korea; 2 Center for Respiratory Safety Research, Division of Jeonbuk Advanced Bio Research, Korea Institute of Toxicology, Jeonbuk-do, Republic of Korea

**Keywords:** radiotherapy, breast cancer, Vactosertib, tumor microenvironment, fibrosis, inflammation

## Abstract

**Background:**

Radiotherapy (RT) is a standard treatment for breast cancer; however, it can induce unfavorable alterations in the tumor microenvironment (TME), including inflammatory responses, fibrosis, and immune dysregulation, which may compromise therapeutic efficacy and contribute to suboptimal treatment outcomes. This study aimed to investigate whether Vactosertib, a TGF-β receptor I inhibitor, could modulate RT-induced TME changes and improve treatment outcomes.

**Materials and methods:**

A breast tumor-bearing mouse model was established and treated with RT alone or in combination with Vactosertib. Mice received fractionated irradiation (4 Gy/day for 3 days) and oral administration of Vactosertib (2.5 mg/kg for 2 weeks). Tumor progression, gene expression, cytokine profiles, immune cell infiltration, and fibrosis were analyzed using microarray analysis, quantitative RT-PCR, ELISA, and histological assessments.

**Results:**

RT alone reduced tumor volume but induced transcriptional and cytokine changes associated with inflammation and fibrosis within tumor tissues. In contrast, combination treatment with Vactosertib significantly enhanced tumor suppression compared with RT alone. This was accompanied by reduced expression of pro-inflammatory cytokines, decreased collagen deposition, and increased infiltration of CD8+ T cells, indicating a shift toward a more favorable anti-tumor immune microenvironment.

**Conclusions:**

These findings suggest that Vactosertib may enhance the therapeutic efficacy of RT by modulating radiation-induced inflammatory and fibrotic changes in the TME, supporting its potential as a rational combination strategy to improve RT outcomes in breast cancer.

## Introduction

Breast cancer is one of the most prevalent cancers globally, and despite advancements in early diagnosis and treatment strategies, it remains a significant clinical burden.^1^ Radiotherapy (RT) is a core component of breast cancer treatment, and it is estimated that approximately 60–70% of all patients receive RT during their course of treatment.^2^ In particular, it has become a standard treatment with proven effectiveness in reducing local recurrence and improving clinical prognosis after breast-conserving surgery.^3^

However, recent studies suggest that the therapeutic benefits of RT may be accompanied by unintended changes in the tumor microenvironment (TME).^4^ Radiation induces not only direct cytotoxic effects on tumor cells but also various biological changes such as the production of inflammatory cytokines, extracellular matrix (ECM) reorganization, and dysregulation of immune responses.^4,5^ In particular, ECM reorganization, including collagen accumulation and fibrosis, can limit effective anti-tumor immune responses by increasing tissue stiffness and inhibiting immune cell infiltration.^4,6^ In addition, severe radiation-induced toxicity can lead to the discontinuation or dose reduction of treatment, which can ultimately reduce the overall effectiveness of RT.^7^ These changes may act as important factors that impair treatment responsiveness by remodeling the TME, rather than directly inducing tumor recurrence or metastasis.^4,5,7^

These changes are regulated by various signaling pathways, among which the Transforming growth factor-β (TGF-β) signaling pathway is a well-established regulator implicated in TME remodeling.^8,9^ Activation of TGF-β signaling after radiation exposure is closely associated with increased accumulation of ECM components, changes in inflammatory cytokine networks, and suppression of anti-tumor immune responses.^10^ In particular, TGF-β signaling can act as an upstream regulator of multiple pathological processes, such as fibrosis, immunosuppression, and ECM reorganization.^8,9^ Therefore, unlike conventional anti-inflammatory strategies that target individual cytokines, approaches targeting TGF-β offer the potential to regulate complex changes more comprehensively within the TME.^11,12^

In this context, Vactosertib, a selective TGF-β receptor 1 inhibitor, is emerging as a promising therapeutic agent in clinical and non-clinical studies.^13,14^ Previous studies, particularly those conducted by our research group, have reported that inhibition of TGF-β signaling with Vactosertib can effectively inhibit tumor progression without significant systemic toxicity.^15–17^ These characteristics suggest that Vactosertib has the potential to be utilized as a combination strategy to modulate radiation-induced alterations in the TME and enhance therapeutic efficacy.

Despite increasing interest in various strategies to mitigate RT-induced toxicity, how key biological changes within the TME, such as immune responses, inflammatory responses, and fibrosis, are regulated when RT is combined with targeted therapies has not yet been sufficiently elucidated. Therefore, this study investigated whether combination treatment with Vactosertib and RT could modulate radiation-induced TME alterations and improve therapeutic outcomes, focusing on inflammatory cytokines, immune infiltration, and fibrosis.

## Materials and methods

### Chemicals

Vactosertib (EW-7197) was obtained from Dr. D.K. Kim of Ewha Womans University (Seoul, Republic of Korea).

### Cell culture

4T1-luc mouse mammary tumor-derived cells were obtained from ATCC and cultured in Dulbecco’s Modified Eagle Medium supplemented with 5% fetal bovine serum and penicillin–streptomycin. Dulbecco’s Modified Eagle Medium and fetal bovine serum were purchased from GenDEPOT (Katy, TX, USA), and penicillin–streptomycin was obtained from the same supplier. Cells were maintained at 37°C in a humidified incubator with 5% CO_2_.

### Animals

Four-week-old female BALB/c mice (Central Lab. Animal Inc., Seoul, Republic of Korea) were housed under specific pathogen-free conditions with *ad libitum* access to sterile food and water. The animal facility was maintained at 21°C with 50% humidity under a 12-h light/dark cycle.

After a 1-week acclimation period, mice were inoculated with 4T1-luc cells (4 × 10^4^ cells) into the fourth mammary fat pad. When tumor volumes reached approximately 100 mm^3^, mice were randomly assigned to three groups (n = 8 per group): control (no treatment), radiotherapy alone (RT), and radiotherapy plus Vactosertib (RT + Vac).

Whole-body irradiation was administered at a dose of 4 Gy per day for three consecutive days. This fractionated regimen was selected to induce measurable tumor and microenvironmental responses while minimizing excessive toxicity, based on commonly used preclinical irradiation protocols. Vactosertib (2.5 mg/kg/day) was administered via oral gavage for 2 weeks, 30 min prior to irradiation, based on previously reported preclinical protocols and effective dosing regimens.^15,17^ Body weight was measured twice a week during the experiment period to monitor potential systemic toxicity.

All animal experiments were approved by the Institutional Animal Care and Use Committee of Ewha Womans University and conducted in accordance with the NIH Guide for the Care and Use of Laboratory Animals.

### Microarray analysis

Total RNA was isolated from mammary tumor tissues using TRIzol™ reagent (Thermo Fisher Scientific, Waltham, MA, USA) and purified using the RNeasy kit (Qiagen, Germany). RNA samples were analyzed using the Affymetrix GeneChip Mouse Gene 2.0 ST array and scanned with the Affymetrix GeneChip™ scanner. Data were normalized, and differentially expressed genes (DEGs) were identified based on fold change ≥ 2 and a p-value < 0.05.

### qRT-PCR analysis

cDNA was synthesized from 1 μg of total RNA using M-MLV reverse transcriptase and random primers. Quantitative real-time PCR was performed using SYBR Green dye and a StepOnePlus™ Real-Time PCR system (Applied Biosystems, Foster City, CA, USA).

### Immunofluorescence assay

Tumor sections were fixed with 4% formaldehyde and blocked with 5% bovine serum albumin containing Triton X-100. Sections were incubated with anti-CD8a (PE, BD Biosciences, San Diego, CA, USA) at 4°C overnight. Nuclei were counterstained with DAPI, and fluorescence signals were visualized using a fluorescence microscope (Axio Observer, Carl Zeiss, Oberkochen, Germany).

### Masson’s trichrome staining

Tumor tissues were fixed in 4% formaldehyde, embedded in paraffin, and sectioned. Sections were deparaffinized, rehydrated, and stained using standard Masson’s trichrome staining procedures to evaluate collagen deposition.

### Cytokine measurement

Levels of IL-4, IL-13, IL-17A, vascular endothelial growth factor (VEGF), tumor necrosis factor-α (TNF-α), IL-23p19, macrophage colony-stimulating factor (M-CSF), granulocyte-macrophage colony-stimulating factor (GM-CSF), CCL11, and CXCL12 in tumor lysates were measured using ELISA kits (R&D Systems, Minneapolis, MN, USA) according to the manufacturer’s instructions.

### Statistical analysis

Data are presented as mean ± standard error of mean (SEM). Statistical significance was determined using one-way analysis of variance (ANOVA) followed by Tukey’s post hoc test. Analyses were performed using GraphPad Prism software. A p-value < 0.05 was considered statistically significant.

## Results

### Effects of radiotherapy (RT) and Vactosertib on tumor microenvironment (TME)

**FIGURE 1.**
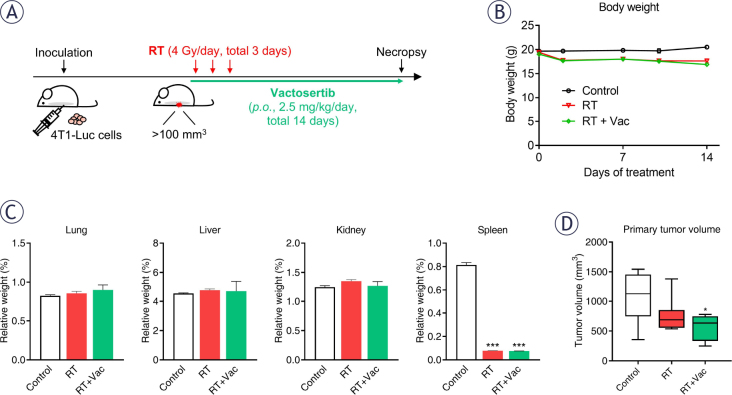
**Experimental design and treatment effects of RT and Vactosertib in a breast cancer mouse model. (A)** Schematic overview of experimental design. BALB/c mice bearing 4T1-luc mammary tumors (~100 mm^3^) were randomly assigned to control, radiotherapy (RT), or radiotherapy plus Vactosertib (RT + Vac) groups. Mice received whole-body irradiation (4 Gy/day for 3 consecutive days) with or without Vactosertib treatment, followed by molecular and histological analyses including gene expression profiling. **(B)** Body weight changes during the treatment period (0, 2, 7, 10 and 14 days). No significant differences were observed among the groups. **(C)** Relative organ weights of lung, liver, kidney, and spleen at the endpoint. Spleen weight was significantly reduced in RT-treated groups compared with that in the control group. **(D)** Tumor volume at day 14 after treatment. Combination treatment (RT + Vac) resulted in greater tumor growth inhibition compared with RT alone. Data are presented as mean ± standard error of mean (SEM) (n = 8 per group). Statistical significance was determined using one-way analysis of variance (ANOVA) followed by Tukey’s post hoc test (*p < 0.05, ***p < 0.001).

To investigate the differences in tumor gene expression following RT and Vactosertib treatment, we generated mouse models receiving RT alone or in combination with Vactosertib pretreatment (Figure 1A). The body weight of RT-treated mice was slightly lower than that of the control group; however, no statistically significant difference was observed (Figure 1B). Additionally, RT and Vactosertib did not significantly affect the weights of the lung, liver, and kidney, whereas spleen weight was significantly reduced in RT-treated mice (Figure 1C). Moreover, although RT alone reduced mammary tumor volume, the combined treatment further enhanced tumor suppression compared with RT alone (Figure 1D).

**FIGURE 2.**
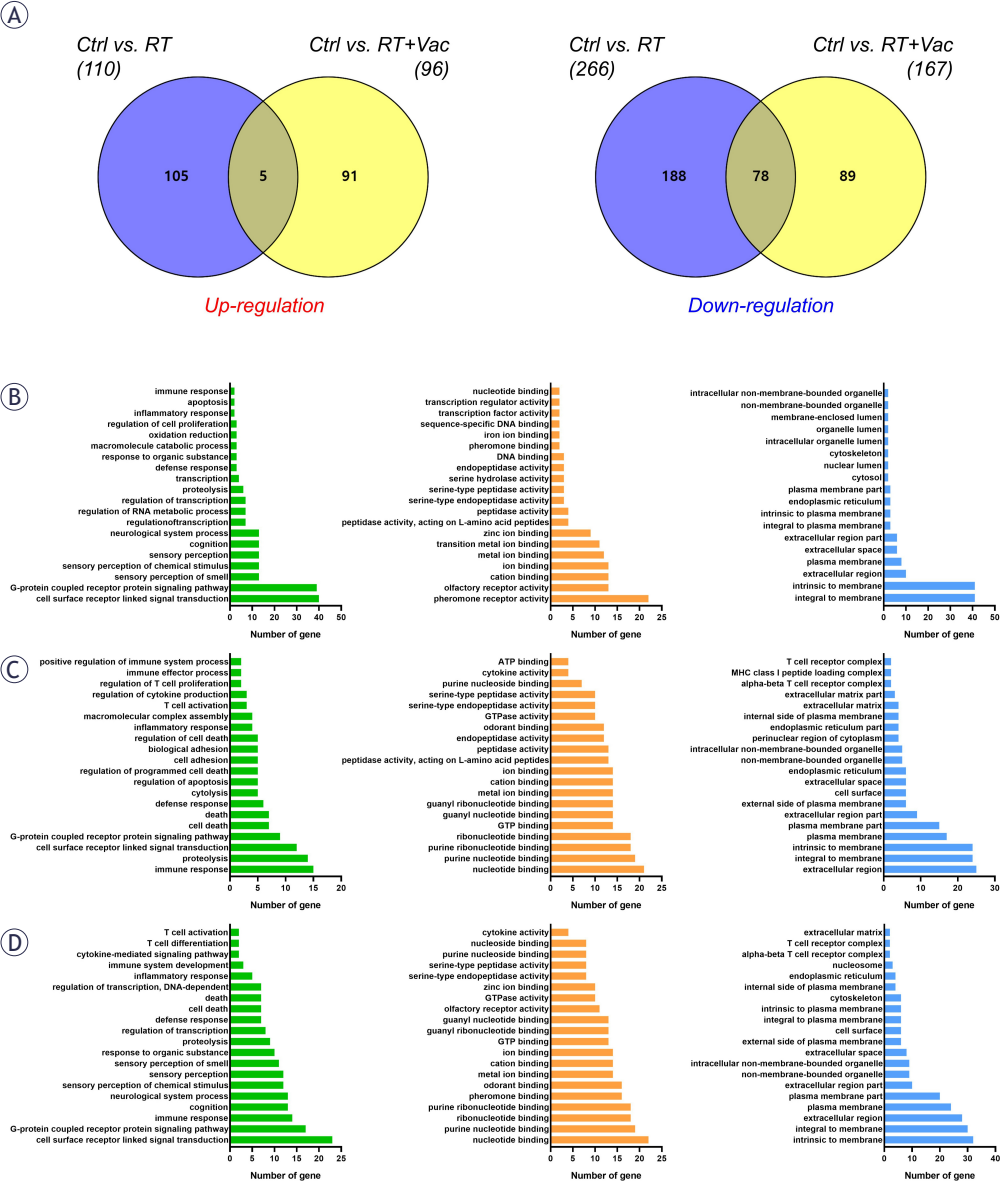
**Transcriptomic profiling and Gene Ontology (GO) functional enrichment analysis of differentially expressed genes. (A)** Venn diagrams showing the number of unique and overlapping up-regulated (left, red) and down-regulated (right, blue) differentially expressed genes (DEGs) in tumor tissues from the radiotherapy (RT) and radiotherapy plus Vactosertib (RT + Vac) groups compared with the Control group (|FC|≥2, 2, p < 0.05). **(B-D)** GO enrichment analysis of DEGs categorized into Biological Process (green), Molecular Function (orange), and Cellular Component (blue). Panels represent enriched terms for the comparisons of **(B)** Control vs. RT, **(C)** Control vs. RT+Vac, and **(D)** RT vs. RT+Vac.

We next performed microarray analysis using tumor samples from each group to identify differentially expressed genes (DEGs). A total of 110 and 26 genes were up-regulated and down-regulated, respectively, in the RT group compared with the control group. In the RT plus Vactosertib group, 96 genes were up-regulated, and 167 genes were down-regulated (Figure 2A). These results indicate that the combined treatment induced broader transcriptional reprogramming compared with RT alone.

DEGs were further analyzed using Gene Ontology (GO) analysis through the DAVID database. The top 20 enriched GO terms in biological processes (BPs), molecular functions (MFs), and cellular components (CCs) are presented (Figure 2B-C). Notably, BPs associated with immune system processes, inflammatory response, and T cell activation were highly enriched in the combination treatment group compared with the control and RT groups. In addition, MFs related to cytokine activity and CCs associated with the T cell receptor complex were prominently enriched (Figure 2B-D). These findings suggest that Vactosertib induces transcriptional reprogramming associated with immune-related processes.

### Vactosertib improves response to RT-induced tumor microenvironmental changes

**FIGURE 3.**
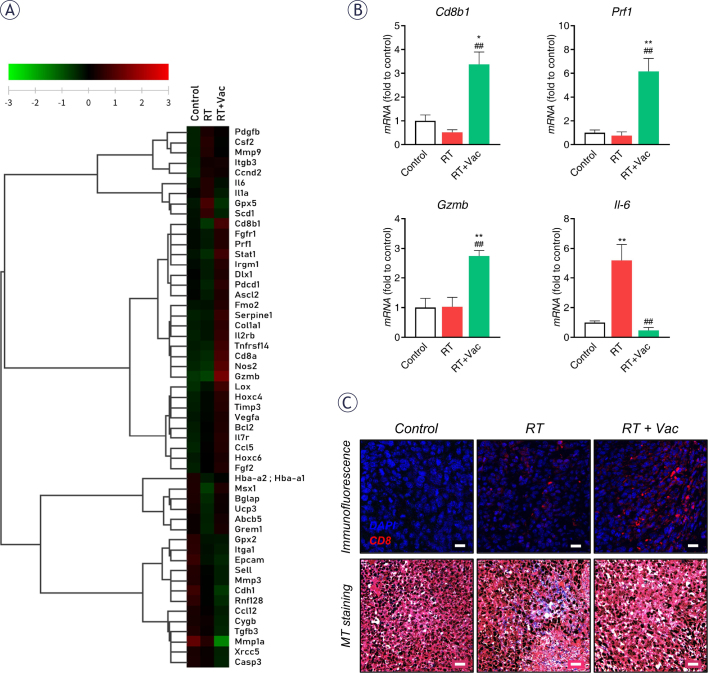
**Enhancement of anti-tumor immunity and modulation of the TME by the combination of RT and Vactosertib. (A)** Heatmap analysis showing the differential expression of selected genes involved in immune response, apoptosis, and tissue remodeling in 4T1-luc tumors from Control, Radiotherapy (RT), and Radiotherapy plus Vactosertib (RT + Vac) groups. Red and green colors indicate up-regulated and down-regulated genes, respectively. **(B)** qRT-PCR validation of key immune-related genes, including *Cd8bl, Gzmb, Prfl*, and *Il-6*. The mRNA expression levels were normalized to those of the Control group. Data are presented as mean ± standard error of mean (SEM) (n = 8 per group). Statistical significance was determined using one-way analysis of variance (ANOVA) followed by Tukey’s post hoc test (*p < 0.05, **p < 0.01 vs. Control; ##p < 0.01 vs. RT). **(C)** Representative histological images of tumor sections. (Upper) Immunofluorescence staining for CD8 (red) and DAPI (blue) showing the infiltration of CD8+ T cells into the tumor tissue. Scale bar = 20 μm. (Lower) Masson’s trichrome (MT) staining for evaluating collagen deposition and tissue morphology within the TME. Scale bar = 20 μm.

GO analysis indicated that both RT and Vactosertib are associated with tumor immunity. To further identify key genes involved in these processes, we generated a heatmap based on gene clustering of selected DEGs (Figure 3A). Genes associated with oxidative stress, DNA repair, apoptosis, T cell anergy and immune tolerance, fibrosis, and stemness were differentially regulated across the treatment groups.

Based on these findings, quantitative RT-PCR was performed to validate immune-related gene expression in tumor tissues (Figure 3B). Expression levels of *Cd8bl, Gzmb*, and *Prfl* were decreased or unchanged following RT alone but were significantly increased in the combination treatment group. In contrast, *II-6* expression was significantly elevated following RT but was markedly reduced by the addition of Vactosertib.

To further evaluate immune responses, immunofluorescence analysis was conducted to assess CD8^+^ T cell infiltration in tumor tissues. RT alone slightly increased the CD8 signal intensity compared with the control group.

In addition, histological analysis was performed to assess fibrosis-related changes. RT significantly increased the collagen deposition within tumor tissues, whereas Vactosertib combination treatment attenuated RT-induced collagen accumulation (Figure 3C). These findings are consistent with transcriptomic alterations observed in genes related to immune regulation and fibrosis.

### Vactosertib modulates RT-induced cytokine expression

**FIGURE 4.**
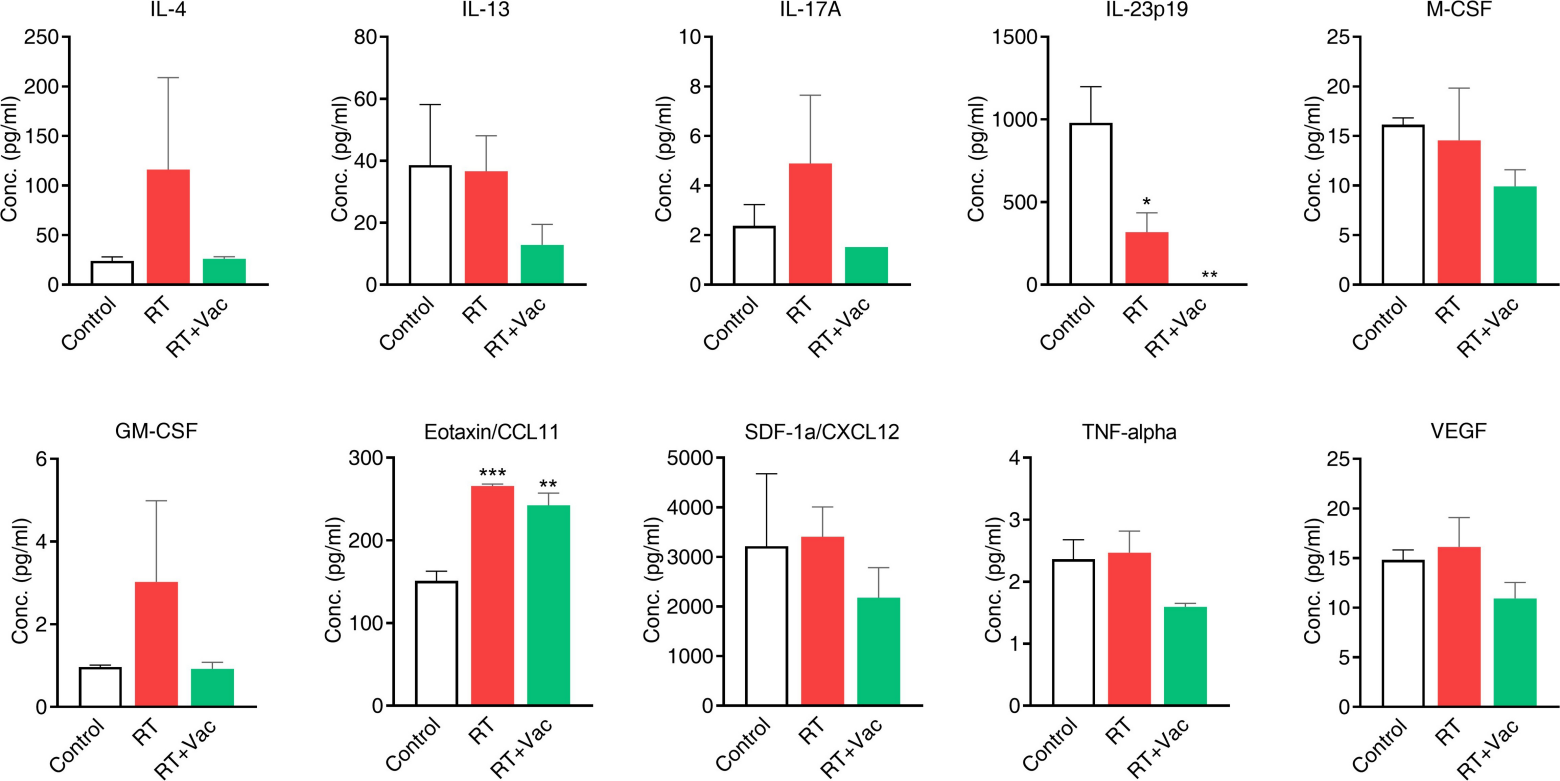
**Impact of RT and Vactosertib on cytokine and chemokine profiles in the TME**. The concentrations of various cytokines and chemokines, including IL-4, IL-13, IL-17A, IL-23pl9, M-CSF, GM-CSF, Eotaxin/CCL11, SDF-1a/CXCL12, TNF-alpha, and VEGF, were measured in tumor lysates using ELISA. 4T1-luc tumor-bearing mice were treated with Control (white), Radiotherapy (RT, red), or Radiotherapy plus Vactosertib (RT + Vac, green). Data are expressed as mean ± standard error of mean (SEM) (n = 8 per group). Statistical significance was determined by one-way analysis of variance (ANOVA) followed by Tukey’s post hoc test (*p < 0.05, **p < 0.01, ***p < 0.001).

We next analyzed cytokine expression levels to evaluate changes in inflammatory signaling within the TME (Figure 4). RT increased the expression of IL-4, IL-17A, VEGF, TNF-α, GM-CSF, CCL11, and CXCL12, whereas these cytokines were reduced following combination treatment with Vactosertib.

Additionally, IL-13, IL-23, and M-CSF levels showed a decreasing trend following RT and were further reduced by combination treatment, although these results were not significant. Overall, a consistent trend toward reduced inflammatory cytokine expression was observed in the combination treatment group compared with RT alone. These results suggest that Vactosertib exerts anti-tumor effects, at least in part, by suppressing radiation-induced inflammatory responses within the TME.

## Discussion

This study demonstrated that combination therapy with RT and Vactosertib can enhance antitumor efficacy by modulating changes in the radiation-induced TME in a breast cancer model. Specifically, compared to RT alone, Vactosertib combination therapy suppressed inflammatory cytokine expression, reduced fibrosis, and increased cytotoxic T cell responses, suggesting the potential therapeutic effects of this combination therapy.

RT is widely used as a standard treatment for breast cancer due to its effectiveness in suppressing tumor recurrence and local control.^2,3,18^ However, it can induce various biological responses within the TME. In this study, RT alone resulted in increased expression of inflammatory cytokines and fibrosis-related changes, including collagen deposition, which is consistent with previous findings on radiation-induced tissue remodeling and inflammatory activation.^4,15^ These changes can contribute to an adverse TME by inducing immune dysregulation and inhibiting effective anti-tumor immune responses.^4,5^ In addition, radiation can induce the accumulation of immunosuppressive cells or the decline in T cell function within the TME, potentially limiting the sustainability of the anti-tumor immune response.^19^

In particular, this study confirmed that when Vactosertib was used in combination with RT, immune activation was enhanced through increased CD8+ T cell infiltration and increased expression of cytotoxic markers such as granzyme B and perforin. Although a Vactosertib monotherapy group was not included in this study, previous reports have demonstrated that Vactosertib alone exerts minimal tumor growth inhibition in the 4T1 model under comparable conditions.^16^ Therefore, the enhanced tumor suppression observed in the combination group is likely attributable to modulation of radiation-induced tumor microenvironmental changes rather than a standalone cytotoxic effect of Vactosertib. In contrast, RT monotherapy showed limited or inconsistent effects on these immune markers. These results suggest that while RT can induce partial immune activation, it may be limited in inducing a sufficient anti-tumor immune response without additional modulation of the TME.5 These limitations may also be related to the immunosuppressive cytokine environment and the decline in immune cell function.

Furthermore, cytokine profiling results showed that RT tended to increase the expression of various inflammatory and tumor-promoting cytokines, including IL-6, TNF-α, and Th2-related cytokines. These cytokines are closely associated with tumor progression, immune evasion, and fibrosis.^20^ In particular, IL-6 is known to promote tumor survival and immune evasion by activating STAT3 signaling, and TNF-α can contribute to tumor progression by creating a chronic inflammatory environment.^21,22^ In addition, Th2-related cytokines have been reported to be involved in shifting the TME to an immunosuppressive state.^23,24^ After combination therapy with Vactosertib, the expression of these cytokines showed an overall decreasing trend, although not all changes reached statistical significance, suggesting that modulation of inflammatory signaling may partly contribute to the observed therapeutic efficacy. These results comprehensively expand upon previous findings regarding radiation-induced inflammation or TGF-β inhibition. Our findings indicate that combining these treatments may act not only through isolated pathways, but also by regulating the TME as a whole.

Vactosertib is a selective inhibitor of TGF-β receptor 1 and has been reported to exhibit antitumor, antifibrotic, and immunomodulatory effects in various cancers.^25–28^ These findings are consistent with the known downstream effects of TGF-β signaling inhibition, as previously demonstrated in our earlier study showing suppression of SMAD2/3 phosphorylation by Vactosertib.^15,17^ Although this study did not directly evaluate TGF-β signaling activity, the observed reduction in fibrosis, inhibition of inflammatory cytokines, and increase in cytotoxic T cell responses can be interpreted as consistent with existing TGF-β inhibitory effects.^9^ In particular, TGF-β signaling is known to inhibit CD8+ T cell infiltration and promote immune evasion in the TME^11,12^, so strategies to inhibit it can contribute to restoring the anti-tumor immune response. However, additional mechanistic studies are needed to confirm whether these effects are directly mediated by TGF-β signaling inhibition.

This study has a few limitations that should be considered. First, our mechanistic analysis focused on phenotypic changes rather than direct TGF-β signaling activity. This clearly demonstrates the response of TME, but further research to elucidate the molecular biological mechanism will provide more precise information. Second, we did not include a Vactosertib monotherapy group because the study was specifically designed to investigate its modulatory effects on radiation-induced changes. Given that the individual effects of Vactosertib have been well-documented in prior studies, we built our experimental design on that existing data to focus specifically on how it interacts with RT. While this limits the evaluation of the drug’s standalone impact in this particular setup, it allowed us to more effectively analyze the synergistic potential of the combination therapy. Third, we used whole-body irradiation instead of localized treatment to ensure that all experimental animals received a uniform dose. While localized irradiation is closer to clinical practice, our approach helped maintain consistency in observing the TME across different subjects. We acknowledge that systemic immune responses from whole-body irradiation might overlap with tumor-specific effects, and this should be further validated in localized models. These points provide a clear direction for future research to build on our current findings.

In conclusion, the combination of RT and Vactosertib can enhance therapeutic efficacy by alleviating radiation-induced inflammation and fibrosis within the TME and associated with a more favorable immune microenvironment characterized by increased CD8+ T cell infiltration. This combination strategy can be considered an adjuvant therapy approach with potential translational relevance, capable of complementing the limitations of the TME while maintaining the efficacy of RT.
